# A Learning Healthcare System for pregnant and breastfeeding women: what do women during preconception, pregnancy, and nursing think? – A qualitative study: A contribution from the ConcePTION project

**DOI:** 10.1186/s12884-022-04675-2

**Published:** 2022-04-18

**Authors:** Marieke J. Hollestelle, Rieke van der Graaf, Sarah Dewi Hartman, Miriam C. J. M. Sturkenboom, Johannes J. M. van Delden

**Affiliations:** 1 Department of Medical Humanities, Julius Center for Health Sciences and Primary Care, University Medical Center Utrecht, Utrecht University, Utrecht, the Netherlands; 2 Department Data science & Biostatistics, Julius Center for Health Sciences and Primary Care, University Medical Center Utrecht, Utrecht University, Utrecht, the Netherlands

**Keywords:** Ethics, Qualitative research, Learning healthcare systems, Pregnant and breastfeeding women

## Abstract

**Background:**

Most medications lack evidence-based information about its safety and efficacy during pregnancy and breastfeeding, because pregnant women are often not included in clinical research. Another way to generate evidence is by using a Learning Healthcare System (LHS) approach. In an LHS, care and research are aligned in such a way that it can accelerate evidence generation and outcomes for patients, based on real-life medication use. For the development of an ethically responsible and sustainable LHS, it is of crucial importance to understand what women think of such an alternative approach to knowledge generation. Therefore, this paper explores their views on an LHS for pregnant and breastfeeding women.

**Method:**

For this qualitative study, we interviewed 20 women during preconception, pregnancy, or nursing to explore their views on an ethically responsible LHS for pregnant and breastfeeding women. The pseudonymized transcripts were analyzed thematically.

**Results:**

We identified four main themes describing women’s views on LHSs. The first theme describes that respondents were positive about learning healthcare systems, and considered them to function as a central point for information about their medication, which they felt is currently lacking. The second theme shows that respondents want to contribute to and engage in generating new information because they want to help others and contribute to scientific research. Respondents also mentioned that, currently, not every woman is aware of the risks of the lack of evidence for medication used in pregnancy. The third theme shows that respondents regard their healthcare professional as essential for the translation and interpretation of information, regardless of a learning healthcare system. The last theme describes that respondents will trust a learning healthcare system more if the medical community supports it, and when data collection and processing is transparent.

**Conclusion:**

Women during preconception, pregnancy and nursing agree that an LHS could be a viable alternative to help close the knowledge gap on the safety of medication used during pregnancy and breastfeeding. The obtained insights from our interviews provide valuable stepping-stones for the development of an ethically responsible and sustainable LHS, as well as for the engagement of women in an LHS.

**Supplementary Information:**

The online version contains supplementary material available at 10.1186/s12884-022-04675-2.

## Background

Every year more than 5 million women^1^ become pregnant in the European Union and the majority takes at least one medication during a pregnancy [1, 2]. Yet, most medications lack evidence-based information about safety and efficacy during pregnancy, because pregnant women are routinely excluded from most clinical research, due to a fear of harming the developing fetus [3, 4]. Even less information is available about the exposure of the newborn to the medication through breastfeeding. Only 5% of the available medications have been adequately monitored, tested, and labelled for use in pregnancy and breastfeeding and often long-term effects remain unknown [5].

In real life, numerous medications have been used safely and effectively in pregnancy with minimal risk to the fetus and mother, but we are not systematically learning from these experiences [6, 7]. There are strong ethical reasons to change the way evidence is currently being generated and disseminated. In the literature, multiple solutions for conducting research with pregnant women have been suggested, such as, routine inclusion of pregnant women in clinical trials or using an adaptive trail design to support safe and efficient inclusion of pregnant women in different stages of medication development [3, 8]. However, pregnant women hesitate to participate in trials, and medicines manufacturers hesitate to have them included, because of potential liability issues. Given the vast availability of real-world data on medicines prescriptions and health outcomes, an alternative way to generate evidence is to learn from previous and current medication use, by transforming the field of pregnant and breastfeeding women into a Learning Healthcare System (LHS) [4]. In an LHS, healthcare and research are aligned to accelerate research and outcomes for patients. LHSs have the potential to develop scientific knowledge based on health information and research data, and by directly implementing new insights from analyses to the clinical practice [9].

Currently, information on the safety and efficacy of medications used during pregnancy and breastfeeding is fragmented and spread across different data sources, pregnancy or medicines cohorts, registries, and research groups with unique data regarding pregnancies, adverse drug reactions and the like. Examples are the European system for the evaluation of safety of medication use in pregnancy in relation to risk of congenital anomalies (EUROmediCAT) and the European Network of Teratology Information Services (ENTIS). Combining these unique data sources in a system of continuous learning could help clarify how medications impact pregnancy outcomes and breastfeeding exposures [4, 7].

Although an LHS approach may broaden the opportunities to strengthen the evidence base of medications used during pregnancies and breastfeeding, multiple ethical issues arise when establishing and sustaining an LHS [10]. These ethical issues are for a large part the result of the sharp distinction that is currently visible between research and practice. In general, there is the question of quality and usability of the results from the learning activities flowing from an LHS, and therefore, the classification of the learning activities as (scientific) research. Furthermore, LHSs might struggle with ethical oversight, especially when the boundary between research and care is becoming less clear. Other important issues involve notifying participants and asking informed consent, creating transparency regarding data analyses, commercial interests, and unintended negative consequences from implementation of new insights into practice [11]. Furthermore, transforming the field of pregnant and breastfeeding women into an LHS will, besides overcoming the ethical issues, also depend on the support of a broad range of key stakeholders within the health system [12]. For example, women need to trust there is significant value and quality in the alternative approach so that they can rely on this evidence, and they need to believe their concerns about this new approach are taken seriously [12]. However, there is not much knowledge of patients’ perspectives, let alone women of childbearing age, on LHSs. Currently, we do not know what their concerns are and when they would trust and support an LHS. Understanding what women think of this alternative approach and what their concerns are, will be of crucial importance for the success of the implementation of new insights into care and the collection of new health-related data within the LHS. Therefore, this paper aims to explore the views of women on an ethically responsible and sustainable LHS for pregnant and breastfeeding women. To deepen our understanding of the views of women whose data may become part of such an LHS for pregnant and breastfeeding women, we conducted semi-structured in-depth interviews with women during preconception, pregnancy, and nursing. During our interviews, we used the Innovative Medicine Initiative (IMI) ConcePTION-project as a case study. ConcePTION aims to develop an LHS mechanism for pregnant and breastfeeding women. In this way, the questions and answers are less hypothetical and can already be placed in real life context.

## Method

### Design

We employed a qualitative study design to explore women’s views on an ethically responsible LHS for pregnant and breastfeeding women. The study is reported in accordance with the consolidated criteria for reporting qualitative studies (COREQ) [13]. This qualitative interview study is a sub study of the IMI ConcePTION-project. Our study focused solely on women, since we were interested in the primary target population of the LHS specifically, which accordingly could be aligned with the opinion of other relevant stakeholders within IMI ConcePTION. For example, other researchers within the ConcePTION-project conducted a survey study and focus groups with healthcare professionals (HCPs) to understand their needs regarding medication use during pregnancy.

We performed semi-structured interviews with a topic list (see Table 1), which came from two sources. We used some of the items from guideline 12 of the 2016 Council for International Organizations of Medical Sciences (CIOMS) International Ethical Guidelines for Health-related Research Involving Humans [14]. The CIOMS guideline 12 covers essential elements for governing the collection, storage, and analysis of data in health-related research. A parallel can be drawn between data analysis within an LHS and health-related research in general, and therefore, the CIOMS guideline is very relevant for an LHS. We also used the results of a narrative review on patient and public views and attitudes towards the sharing of health data for research [15]. This review gave insight into key conditions for the use of health data in general, which were used as topics in the interviews.

**Table 1:** General topic list

1. Attitude towards the status quo and the goal of ConcePTION	
2. Participatory engagement	
3. Respect for autonomy	
4. Perceived risks	
5. Need for return of results	
6. Inclusion and freeriding	
7. Sustainability	

### Sample and setting

We aimed to include women whose data may become part of an LHS for pregnant and breastfeeding women. We therefore included women who wanted to become pregnant, were pregnant at the time of the interview, or were nursing^2^ up to 6 months after giving birth. Furthermore, to obtain a broad range of perspectives on the topics, we aimed to include women with different medical backgrounds and diverse characteristics. Respondents were recruited by purposeful sampling with the help of our contact persons from the University Medical Center Utrecht, the Amsterdam University Medical Center, The Netherlands Pharmacovigilance Center Lareb, Eurocat Northern Netherlands, and by means of snowball sampling. Potential respondents were then approached and informed about the set-up of the study by e-mail or by phone.

Since an effective LHS for the treatment of pregnant and breastfeeding women is currently lacking, respondents were unfamiliar with the concept of an LHS. To collect valuable answers from the respondents, we decided to give them additional information at the start of the interview. With the additional information, we introduced IMI ConcePTION (see Table 2) as a case study to explain the lack of scientific knowledge and to explain the alternative way to help close the knowledge gap. We assumed all respondents were unfamiliar with the concept of an LHS, and therefore choose to explain the approach ConcePTION is taking and further explained the term LHS as an ecosystem of continuous learning from routinely collected health data. During the interviews, the term ecosystem was used to refer to an LHS, since an LHS is mostly an academic term. In this paper, we will use the term LHS, since it is a commonly used term for systems of continuous learning in healthcare settings in the medical literature. To further explain and to help visualize all the different components of the ConcePTION ecosystem, we used a diagram (see supplementary file 1). The diagram allowed us to emphasize the circular flow in an LHS and to show the important steps in an LHS: data collection, analysis and interpretation, and output. After this short introduction, we started with the first two7 topics. Once these topics were discussed, we made sure the respondents understood what was meant with health data and explained how currently in the Netherlands data is being collected, stored, and used. Then we continued with the rest of the interview.

**Table 2:** IMI-ConcePTION

In April 2019, the Innovative Medicines Initiative (IMI) launched the ConcePTION project (Continuum of Evidence from Pregnancy Exposures, Reproductive Toxicology and Breastfeeding to Improve Outcomes Now). ConcePTION is a European public-private partnership that aims to develop a Learning Healthcare System (called “an ecosystem”) that can generate and disseminate reliable evidence-based information about medication safety and efficacy during pregnancy and breastfeeding by learning from routinely collected data and research data across Europe [7]. During the interviews we introduced ConcePTION, and made a distinction between ConcePTION as a five-year project, which aims to build a system of continuous learning, and ConcePTION as a sustainable ecosystem, which can eventually share new scientific knowledge. A way of sharing new knowledge is through a knowledge bank, which ConcePTION aims to build for both women and their HCPs [7].	

### Data collection

The semi-structured interviews were conducted by MH (trained qualitative researcher, female, MA, PhD candidate) with a topic list. The topic list was refined after two pilot interviews. According to the technique of constant comparative analysis, the interview topics evolved as the interviews progressed alongside the data analysis [16]. Data was collected from February 2020 to January 2021. In 19 out of 20 interviews, there had been no previous contact between the interviewer and the respondents beforehand. In 1 out of 20 interviews, the interviewer and the respondent had met each other prior to the interview in an informal setting. Five interviews were performed in person in rooms at the UMC Utrecht or at the respondent’s home. Due to Covid-19 restrictions, 15 interviews took place via a secure online platform. The interviews took 41 to 94 min with a median duration of 64 min. During the interviews, the order of questions was adapted to the narrative flow and the openness of the individual respondent. During and after the interviews, MH made notes to enhance the data and to provide a clear context for data analysis. The interviews were audiotaped and transcribed verbatim, coded and stored anonymously. Written consent was obtained from all respondents. Because no intervention was imposed on the participants, the Medical Research Ethics Committee (MREC) Utrecht determined that the study was exempt from ethics review under Dutch law.

### Data analysis

After transcription, we analyzed the interviews according to the thematic analysis method and by going back and forth between data collection and analysis to develop codes [16]. MH coded the transcripts using software program NVivo 12. The interpretations and suitability of the codes were discussed and compared amongst the research team. During analysis, codes were adapted and combined, and new codes were added to the coding list where necessary. A meaning pattern was identified across the data set, leading to the formulation of higher order themes. To enhance the validity of our results, an intern, SDH (female medical student, BSc) read the full transcripts to check the consistency of the thematic framework and critically (re)read the coding list. The findings, including the coding list and formulated higher order themes, were discussed within the complete research team (MH, RG, MS, HD). Furthermore, a member check was executed in the last phase of data analysis to discuss the accuracy and interpretation of our preliminary results [17]. Thematic saturation was reached when the occurrence of new findings ended after 20 interviews.

## Results

Out of the 30 people that were approached, 22 agreed to participate in the study, 2 were excluded, 6 were unable to participate and 2 did not respond. A total of 20 semi-structured interviews were conducted with women who varied in medical indication, stage of pregnancy, and reproductive history. Table 3 shows all relevant characteristics of the respondents.

**Table 3:** Demographic characteristics of the respondents

Respondent	Age	Education	Medical indication	Gravida Para Mater (GPM)^a^	Stage pregnancy
1	31–35	Graduate degree	Chronic condition	G3P0M0	Third trimester
2	31–35	Graduate degree	Chronic condition	G1P1M1	Nursing
3	26–30	Lower vocational (MBO)	Chronic condition	G2P1M1	Second trimester
4	36–40	Graduate degree	Acute condition pregnancy related	G4P1M1	Third trimester
5	31–35	Lower vocational (MBO)	Chronic condition	G3P3M2	Nursing
6	31–35	College (HBO)	Acute condition pregnancy related	G1P1M1	Nursing
7	31–35	Graduate degree	Acute condition pregnancy related	G3P2M2	Nursing
8	26–30	Graduate degree	Healthy	G1P0M0	Second trimester
9	36–40	College (HBO)	Acute condition	G2P2M2	Nursing
10	21–25	Lower vocational (MBO)	Chronic condition	G1P1M1	Wish to become pregnant
11	31–35	College (HBO)	Healthy	G1P0M0	Second trimester
12	36–40	Graduate degree	Anomaly	G1P1M1	nursing
13	41–45	Graduate degree	Healthy	G3P2M0	Third trimester
14	31–35	Graduate degree	Acute condition	G3P2M2	Third trimester
15	31–35	Highschool	Chronic condition	G1G0M0	Second trimester
16	31–35	Graduate degree	Healthy	G3P1M1	Nursing
17	31–35	Graduate degree	Healthy	G2P2M2	Nursing
18	36–40	Lower vocational (MBO)	Chronic condition	G8P0M0	Second trimester
19	41–45	College (HBO)	Chronic condition	G3P1M1	Wish to become pregnant
20	36–40	Graduate degree	Healthy	G2P1M1	Nursing
^a^ Gravida Para Mater (GPM) represents the reproductive history by indicating the number of pregnancies (G), births (P), and children (M) of the respondents					

Based on the interviews we formulated four main themes characterizing women’s views and moral intuitions regarding LHSs. The themes emerged consistently across all interviews. We provide representative quotations to illustrate the themes (see Table 4).

**Table 4:** Representative quotations

**Views on an LHS**	Q1	R13: It is making me happy, the fact that you can merge information from different places to create new knowledge. I get that it is complicated and that you need to think about the methods for analysis and interpretation of results. I think it is a good development, also for the users. In this way, HCPs and women can get unambiguous information.
	Q2	R18: I think [ConcePTION] is very good, because it is just great for future patients and others to easily find good information. […] Because it can be very frustrating right now. […] There is a lot of contradictory and unreliable information on the internet.
	Q3	R4: It is ambitious, because you need to gather a lot of data, you need the right data and the right method for data analysis. Then you also need to interpret results and translate the results into accessible information. Not only in jargon, so that nobody understands the information.
**Willingness to contribute and engage in an LHS**	Q4	R2: For others, yes. [The LHS] is of little use to me, but [contributing] is more to help others in the future.
	Q5	R4: I think it is important that [consent] is asked. And that everything is not just lying around all over the place. Especially when it concerns medical data, I don’t think that’s being careful. So, I think this should be handled with care. Certainly. [..] At least consent should be asked [before data is shared] and it should not be just assumed that people consent to sharing data.
	Q6	R15: I am doing pregnancy-yoga, there I am in a group with all big baby bellies. And I also find it useful that I hear various tips regarding the pregnancy. I like that.
	Q7	R20: I don’t think a lot of people, or pregnant women know that they can contribute to scientific research. If they would know about it, I believe they will contribute. It would help to at least give women information about the possibilities [and about the burdens and benefits of contributing].
**The role of the HCP in an LHS**	Q8	R8: It is better to discuss the interpretation [of results] with a GP or gynecologist. Especially on how does this [medicine] influence me and my body?
	Q9	R3: [regarding medication intake during pregnancy] It depends on the choice you make. That goes for everything in life. You are the one to decide. And if your decision turns out wrong, that mistake is yours not someone else’s.
	Q10	R18: Despite the fact that you can suffer from the same condition, everybody is different, every woman is different, and every pregnancy is different. So, what works for one person, does not necessarily work for the other.
**Trust in an LHS**	Q11	R7: I actually trust that [research] will be conducted in a good and competent way and that my data is being used for scientific research and for improving clinical practice. That would be in line with my own goal, which is nice. So, I do not necessarily need to be informed about every detail of the research process. I don’t think that is problematic.
	Q12	R13: Once there is this additional goal of making profit, you cannot be objective. Even as a researcher you cannot. The pharmaceutical industry can ask researchers for certain results in exchange for a trip to Haiti. In that situation, you are no longer transparent, honest, and objective. Commercial purposes cloud that.
	Q13	R19: It should be promoted by the right people. When I would go to my doctor, for example, my doctor would say to me this is a great website to go to. I go to the midwife and she would say to me this is a great website to go to, etc. I think that’s important.

We started the interviews by asking the respondents about their experiences with the use of medication and with the search for information about their medication. Most respondents mentioned that they experienced difficulties in finding reliable and consistent information and that drug labels lack any useful information on the safety of the medication they wanted or needed to take. We also asked healthy women whether they had taken any medication during pregnancy or breastfeeding. Interestingly, most respondents replied they had not. Only after we asked whether they had looked for information online about medication and we discussed the return of results, did it become apparent that these women had in fact taken multiple medications for milder complications or conditions, during their pregnancy or during birth and/ or recovery.

### Theme 1: views on an LHS

In principle, all respondents expressed a positive attitude towards ConcePTION as a project and as an LHS (Q1). Most respondents considered an LHS to function as a central point for data analysis and/ or as a central point for information. Some respondents emphasized the need for such a central point to help overcome the problem of contradictory information available online or from their HCPs. Some respondents argued that the information that flows from an LHS could increase their confidence regarding the safety and efficacy of medications and would allow them to take control over their own medication intake. They mentioned that they often do not know whether a medication is safe, and therefore, they rather not take any medication at all (Q2).

Some respondents stressed the importance of organizations, experts, HCPs, and patients working together within an LHS. They argued that working together oftentimes means learning from each other through knowledge sharing. Combining knowledge was seen by some of the respondents as an improvement for the generation of new knowledge about the safety and efficacy of medications for, for example, different types of patients, event congenital anomalies, and women in general.

Some respondents immediately addressed the potential risks and hurdles that are associated with large data projects. They argued that they were in favor of collecting health data and the use of their health data in an LHS, as long as their privacy can be protected.

Another initial response of some respondents, was that an LHS is very complicated to understand. Some respondents said that they did not understand how an LHS would work in reality, but argued that it was not up to them to fully grasp it. Furthermore, respondents thought that building an LHS must be very challenging, labor-intensive, and above all highly ambitious, because it involves many stakeholders, and it concerns a lot of data (Q3). A few respondents compared an LHS with big data projects or information technology systems, which according to them, is complex and takes years to set up properly.

### Theme 2: willingness to contribute to an LHS

#### Motivations for contributing

The respondents considered helping other people or helping future generations to be one of the most important reasons to contribute to the development of new information. Respondents emphasized that they want to help with preventing people from experiencing the same struggles they experienced when searching for information on medication and the struggles with becoming pregnant while also dealing with a chronic condition (Q4). Another reason mentioned was to advance scientific research, even if there is no direct benefit for themselves. Respondents highly valued scientific research and argued that it would facilitate the progress in this little explored field.

#### Perceived barriers and facilitators

The respondents emphasized that contributing to the creation of new information within an LHS should be non-invasive and not too time consuming. Examples of invasive and time-consuming contributions mainly had to do with undertaking a complex action, such as having to arrange your own supplies to collect for example milk or urine. Many respondents suggested combining already planned hospital visits, or other pregnancy-related check-ups with research activities to make it more accessible to pregnant and breastfeeding women. Furthermore, most respondents emphasized that the aim of the project or an LHS should be relevant to their own situation, or should be in line with their own health needs and priorities, such as fighting an illness or condition and sharing experiences.

#### Respect for autonomy

Most respondents argued that it is important to at least notify people about collecting and using health data. A small group of respondents wanted to give informed consent for the use of their health data for a study within an LHS. They argued that consent would allow for some control regarding the use of their own data. According to these respondents, data are something personal that should be treated with caution (Q5). Other respondents argued that giving informed consent every time a new study is performed with their data is too invasive and could negatively influence a person’s willingness to contribute. Being (re)contacted for research was sometimes experienced as annoying and was not considered a priority. Furthermore, respondents put forward that when data is anonymous, then there is no added personal value in knowing or giving consent. Furthermore, multiple respondents suggested that when an LHS has been developed it would suffice to have a clear statement on the website explaining how and by whom data is collected, analyzed, and stored. Having information available online allows people to look for the information when they want to know more.

#### Responsibility

All respondents felt a level of responsibility to participate in or contribute to an LHS, if possible. Reasons included: to help prevent other women from experiencing the lack of information about a chronic condition, an adverse drug reaction, the pregnancy, the newborn, doing ‘the right thing’, and helping with research progressing (Q6). Most respondents with a chronic condition explained that they wanted to help other women by sharing their experiences and information, because they felt part of another group or felt connected to other women because of a shared chronic disease or other shared pregnancy or maternal features. Some healthy respondents argued that they did not feel more connected to other pregnant or nursing women and did not need another group to affiliate with and/ or did not want the opinion of other women on how to be pregnant. Some of the healthy respondents also mentioned that the feeling of being connected to other pregnant women was less present during their second pregnancy. A few respondents had the opinion that, unfortunately, some women were not always aware of the knowledge gap, and therefore, do not feel as responsible to participate in research activities (Q7). Further to this, they suggested that more awareness needs to be raised to also reach these women.

### Theme 3: the role of the healthcare professional in an LHS

While the interviews did not specifically focus on the role of the HCPs in the creation of new knowledge, most respondents emphasized the importance of the HCP in both the search for and dissemination of information about medication and treatments while pregnant or breastfeeding.

#### Searching for information

Most respondents found interpreting medical information and research results to be extremely difficult and trying medication by yourself undesirable. Most respondents felt they should consult their HCP (Q8). Many respondents consulted drug labels, the internet, and their HCP for information on the medication they were considering taking. According to them, the internet can be used for personal research prior to a consult or after to read the information again at a slower pace.

#### Dissemination of information

When asked about the return of results in an LHS, most respondents expressed the wish for personalized information. Many respondents valued privacy as an important principle to protect, and therefore, it was acknowledged by a few respondents that personalized information would be difficult to realize without sharing personal information. Respondents mentioned that to fully depend on the information in a knowledge bank, it needs to be able to give decisive advice. Some respondents emphasized the need for a “yes” or “no” answer. Other respondents argued that, if personalized information is not an option, it would still be useful to have information to guide a decision regarding medication intake. A small group of respondents argued that it is always one’s own responsibility to make a good and informed decision (Q9).

Most respondents asked their HCP for advice about the safety and efficacy of medication. Respondents who visit their HCP regularly because of a chronic illness or condition, argued that they rely on their doctors to give them advice on what is desirable for their specific condition. Other respondents emphasized that “everybody is different” and could respond differently to treatment (Q10). Therefore, applying the little information available to one’s own situation is difficult. In general, all respondents trusted their HCP to have the knowledge or to help with deciding what is best for them. Respondents also argued that the HCP probably knows how to interpret the latest news about medication safety, because of their expertise. Some respondents emphasized that in an LHS the benefits for the HCP are much higher in comparison to the direct benefits for themselves. Respondents argued that regardless of an LHS, they would still rather rely on the information from the HCP, because they know more about their specific condition and their context.

### Theme 4: trust in an LHS

#### Trust in research

Most respondents view research as objective, structured and believe there is no conflict of interest. Respondents explained that they trust researchers to handle data correctly and that they trust researchers to follow the rules and regulations regarding data protection. Furthermore, some respondents argued that because they trust researchers, they do not need to be informed about every detail of the research project (Q11).

#### Commercial use and purposes

Commercial use and purposes were also discussed by a group of respondents. Some respondents expressed a cautious or negative attitude towards public-private partnerships in an LHS. Respondents argued that such partnerships could jeopardize the neutrality of the information, since they feel that commercially interested parties’ main objective is to make money (Q12). Some respondents explained that companies like Facebook, news articles on privacy breaches and the negative reputation of the pharma industry make them more cautious of data collection and analysis in general. Respondents emphasized that they would rather not share their personal information with private organizations that make a profit from it. Respondents questioned the level of objectivity of those companies. Respondents expressed that the interference of commercial interests in any system, negatively influences their trust in that system, and therefore, in the information that flows from that system. A small group of respondents argued that although commercial parties have an additional goal, they also stimulate and realize important progress. These respondents expressed a more positive attitude towards collaboration with private organizations in an LHS.

#### Transparency

Most respondents argued that transparency is of great importance for the sustainability of an LHS and for earning their trust in such a system. Respondents explained that to be transparent includes honesty about data collection, data analyses, public-private partnerships, and the way privacy is protected. Transparency also makes the information that flows from it seem more reliable and solid, because it shows that there is “nothing to hide” and all relevant information on how the LHS works is available to anyone who is interested.

#### Broad support from the medical community and the government

Respondents emphasized the need for support of the LHS from the medical community and the government. Having broad support by different authoritative institutions shows that the LHS is well established, and that multiple authoritative people acknowledge and trust the value of the information developed by the LHS. The interviews demonstrated that the respondents considered the research and medical community to be the experts in the assessment of new information, and therefore, respondents rely on their opinion (Q13). Many respondents argued that they would not hesitate using ConcePTION as a source themselves when their HCP would recommend it. Respondents suggested that for ConcePTION to become a sustainable LHS, it should strive to become highly trusted by the medical community.

## Discussion

Our study with 20 women during preconception, pregnancy, or nursing, showed that these women 1) are positive about an LHS for pregnant and breastfeeding women to help diminish the knowledge gap, 2) want to contribute to the development of new information and engage in an LHS, 3) view their HCPs essential in the translation and interpretation of information, regardless of the establishment of an LHS and 4) see trust and transparency as essential for the realization and sustainability of an LHS.

To our knowledge, this is the first study that conducted in-depth interviews with pregnant and nursing women to explore their views on LHSs. In addition to the literature on patients’ and stakeholders’ views on health data research or health information networks, these interviews provide for an extensive understanding of how women view medication intake during pregnancy and breastfeeding, from what perspective women argue for or against contributing to an LHS, and what women of childbearing age need and wish for regarding the return of results in an LHS.

Interestingly, although this is not a quantitative study, all our respondents had taken at least one medication during their pregnancy or during breastfeeding. This finding is in line with what is described in the literature about medication use among pregnant and breastfeeding women [1]. At first, most healthy respondents, who mainly used over-the-counter medication, seemed to think their medication was irrelevant to mention or not as serious compared to medication used for chronic or acute diseases. It seemed that these respondents did not entirely realize that they may be vulnerable when it comes to the risks of a lack of knowledge on medication. At the same time, all respondents experienced difficulties with finding reliable and straightforward information about their medication. These experiences underline the current lack of knowledge and contradicting information, described in the literature [18].

### Solidarity

Earlier studies identified multiple motivators for pregnant women to contribute to clinical research. Similar to our interview study, main motivators are improving medical research, helping others, and having a personal connection to the research subject [19–22]. Interestingly, our respondents also mentioned they felt responsible to contribute and engage to help others with whom they share a specific experience, like having a chronic condition, being in the same stage of the pregnancy, and being a new and young or older mother. In the literature, acting upon this feeling of responsibility to assist others with whom one shares a specific experience, is described as solidarity [23]. Barbara Prainsack and Alena Buyx understand solidarity as a relational practice, where being able to identify with and care for another person in a similar context are of key importance in suggesting new practical solutions to existing problems [23]. Perhaps a solidarity approach in the field of pregnant and breastfeeding women is necessary to include women in the discussion and to allow them to be actively involved in closing the knowledge gap.

Another interesting observation from our study is that women with a chronic condition seemed to experience this personal connection with the research subject and with other women more intensively. A reason for this might be that they already belong to a group of patients with a specific chronic disease or condition. It might, therefore, have been easier for them to picture other women who are going through the same experience of managing their condition and their pregnancy, and they might already have a group of women with whom they share their experiences about having to deal with a chronic disease. Furthermore, their affinity with medical research can possibly be explained by the fact that their pregnancy is medicalized early on [24, 25]. Although, pregnancy and childbirth increasingly have become medically defined phenomena due to medical technology and surveillance focused on risks, women’s experiences with pregnancy-related risks are determined by the interactions with a HCP [25]. Women who suffer from a chronic condition have interactions with their HCP at an early stage, often before their pregnancy. For healthy women, this is probably different, since there are fewer interactions with HCPs and their pregnancy is not fully depended on medical care.

### Dissemination of information

In general, there is a cautious attitude towards medication use during pregnancy or breastfeeding [26]. Our interviews, as well as the literature, showed that women are concerned about the impact of medication on both foetal development and their own health [22, 27]. Our interviews showed that regardless of an LHS, respondents want to know from their HCP whether a medication is safe to use in their situation. The anxiety towards medication use and the difficulty with interpreting medical information, results in a feeling of insecurity [28]. The question is whether an LHS can take away these insecurities. Not only are HCPs important in the dissemination of information among women, but they are also important in the interpretation and translation of new insights that are generated through an LHS. Therefore, the help of HCPs in validating research outcomes and deciding what type of knowledge would be useful to pregnant and breastfeeding women is necessary. Respondents explained they wish to have information that is applicable to their specific situation. It seems that an HCP is of crucial importance in making sure the results generated through an LHS flow back to the patient in understandable language.

Subsequently, pharmaceutical companies have the duty to monitor the safety and efficacy of their medication and to update drug labels once new information becomes available. Unfortunately, it has proven to be extremely difficult to stimulate the progress of updating labels. The European Medicine Agency (EMA) has set up post-authorisation measures (PAM) to make sure drug agencies collect and provide data to enable further assessment on the safety or efficacy of medication in the post-approval setting [29]. Despite these regulations, it still takes too long before labels are updated or the assessments are not completed because of a lack of data [5, 30]. However, providing readable and solid evidence on the safety and efficacy of medication is the task of drug manufacturers. Furthermore, labels are an important source for making an informed decision. Our interviews showed that almost all respondents read the labels before taking any medication during pregnancy or breastfeeding.

### Public-private partnerships and LHSs

Even though we avoided using the term LHS, respondents associated the concept of an LHS mainly with big data, information technology systems, Facebook-like platforms, and medical research in general. Although their associations are not entirely surprising, it did influence their attitude towards ConcePTION as an LHS. The overall negative attitude regarding partnerships with private parties is also often described in the literature as a perceived barrier for sharing health data for research [27, 31]. Individuals seem to be opposed to data sharing if it is motivated by financial gain or profit, or if data is shared with private or commercial companies [31]. To earn the trust of women in an LHS, it seems important to be transparent about the collaboration with private organizations, and to explain why this is vital for the realization and sustainability of an LHS.

### Engagement of pregnant and breastfeeding women

Because there are only a few effective LHSs in practice and because ConcePTION is still an ongoing project, it is not surprising that many respondents did not fully grasp the concept of an LHS. However, for the sustainability and for the willingness of women to engage in an LHS for pregnant and breastfeeding women, it is of crucial importance that women understand what it is, how it works and how certain issues, like privacy, informed consent, and private partnerships are regulated. As Seid et al. (2014) explain, an LHS depends on collaboration and engagement to really improve health care and health outcomes. According to them, engagement can be understood as the extent to which an individual takes part in the generation of new information, knowledge, and know-how, and exists along a continuum ranging from awareness, to participation, to contribution and to ownership of the knowledge generating system [32]. They continue that awareness is the first building block that introduces the individuals with the system and could lead to them becoming participants (using the tools within the system) or eventually contributors (helping with improving the knowledge and resources) [32]. The same could work for women of childbearing age. Meaning that clear information about the LHS, additional tools (sources for more information, research activities, survey studies), and ways for them to be involved (joining a pregnancy advocacy group) need to become available to them. The way to reach women might be, again, different for the group of chronically ill women in comparison to healthy women. As mentioned earlier, women who suffer from a chronic condition, might already be aware of their vulnerable position and might already be involved in patient’s advocacy groups or already participate in research activities.

## Limitations

Our study has a number of limitations. First, we have tried to purposefully include women of all different educational levels, however, we received more responses of highly educated women. Therefore, the possibility of selection bias exists, which challenges the generalizability of the findings. Furthermore, as the results show, we interviewed women who have a positive attitude towards scientific research. This general positive attitude might not be a good reflection of the total population. Second, due to Covid-19 restrictions most of our interviews were held via an online platform instead of face to face. Third, during some of the interviews the subject of privacy was brought up by one of the researchers to help the respondents reflect upon possible risks of an LHS. Bringing up privacy as a possible risk might have altered the answers of the respondents in such a way that privacy became a concern after hearing about it. Fourth, the graph used to visualize the ConcePTION ecosystem was designed with very bright colours. Using these colours might have triggered positive responses to the explanation of the ConcePTION ecosystem, and therefore, the concept of an LHS in general. Fifth, the interviews were conducted with Dutch women only who are in a heterosexual relationship. The Netherlands might reflect a different culture and attitude towards research and health data than other countries. Follow-up research could explore the possible variety of views of women across Europe. In addition, a more inclusive approach is necessary to make sure the (health) interests of all pregnant and breastfeeding people with different sexual orientations and gender identities get equal weight. Despite the limitations of this study, we believe the insights from the study can be used in the development of a sustainable and ethically responsible LHS for pregnant and breastfeeding women.

## Conclusion

To conclude, women during preconception, pregnancy and nursing agree that an LHS could be a viable alternative to generate evidence on medication safety in pregnancy and breastfeeding, which they feel is currently lacking. The obtained insights provide valuable stepping-stones for the development of a sustainable and ethically responsible LHS. Furthermore, the results from this interview study inform the implementation of real-time results flowing from an LHS, as well as encourage the engagement of women in the development of an LHS.

## Supplementary Information

**Additional file 1.**

## Data Availability

The datasets generated and analyzed during the current study are not publicly available because individual privacy could be compromised. Additionally, no permission was asked from the participants for public availability. The dataset is available from the corresponding author on reasonable request.

## Abbreviations

LHS: Learning Healthcare System
HCP: Healthcare Professional
ConcePTION: Continuum of Evidence from Pregnancy Exposures, Reproductive Toxicology and Breastfeeding to Improve Outcomes Now
COREQ: COnsolidated criteria for REporting Qualitative studies

## Footnotes

1 This also includes transgender men, non-binary and gender fluid people who want to become pregnant or are pregnant.

2 We use the word “nursing” instead of “breastfeeding” to respect all different ways women can nurse their newborn, that is for example: breastfeeding, using a breast pump, or using formula milk.
