# Supplementary material for: A Learning Healthcare System for pregnant and breastfeeding women: what do women during preconception, pregnancy, and nursing think? – A qualitative study: A contribution from the ConcePTION project
Source: BMC Pregnancy Childbirth. 2022 Apr 18;22:334. doi: 10.1186/s12884-022-04675-2 (PMC9014975; doi:10.1186/s12884-022-04675-2)
Supplement: Supplementary file 1 — Additional file 1. [file 12884_2022_4675_MOESM1_ESM.docx]

**Supplementary figure 1 ConcePTION Ecosystem**


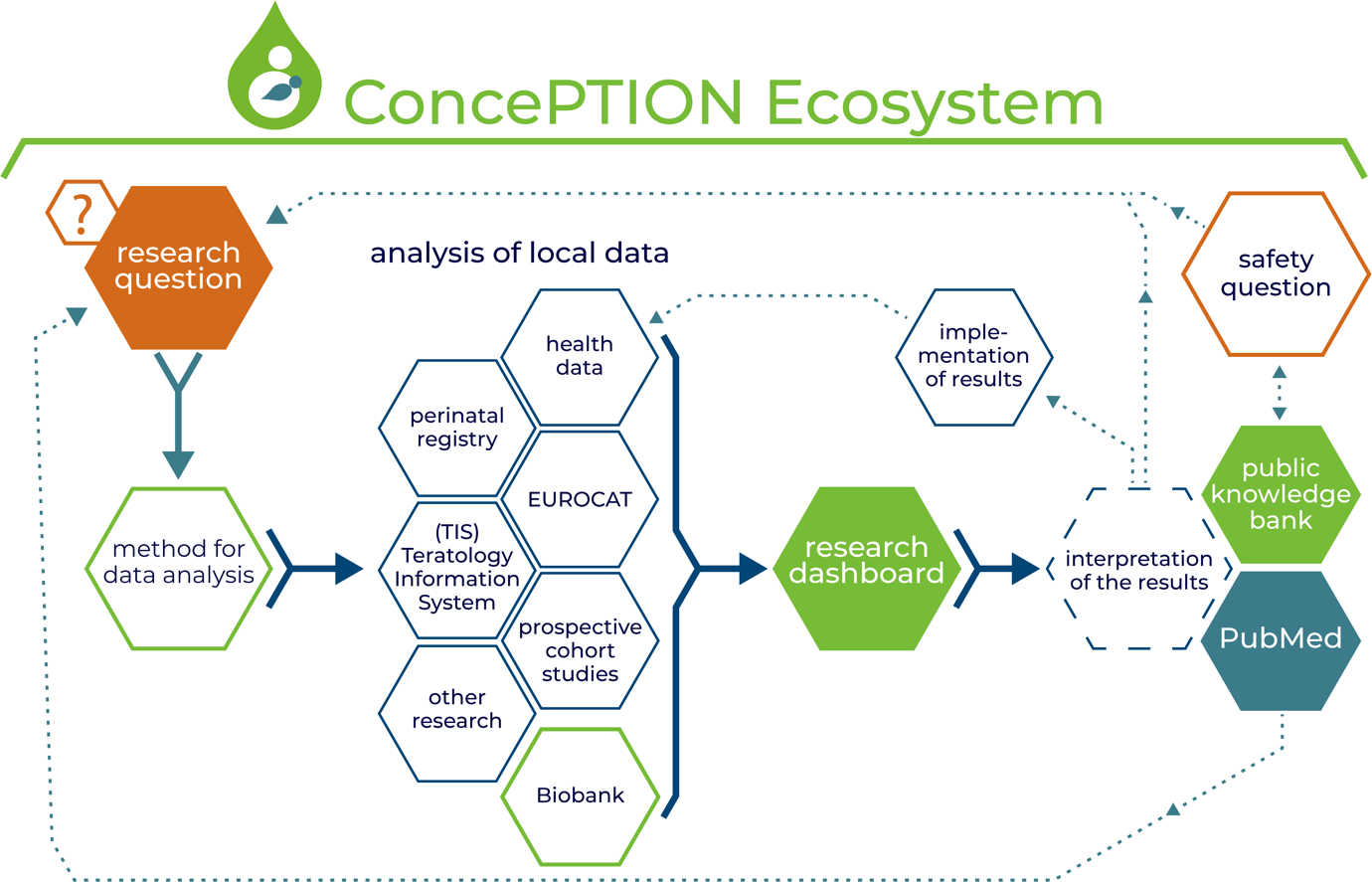
*Designed by STUDIO TERP 2021*

This diagram is based on the graph used during the interviews. The diagram shows the different concepts in the ConcePTION Ecosystem and the different flows within the Ecosystem that emphasize the continuous process of collecting, analyzing, and interpreting data, the implementation of new results in care, and it shows how new results can pose new research questions for which the ConcePTION Ecosystem can be used. In this diagram, the green objects represent the results of the ConcePTION project, namely: a common data model to analyze local data, a Biobank (which will be developed in the future) that collects milk and urine samples for future research on medication exposure, a research dashboard that collects results from the analyses and can be used for the interpretation of results, and a public knowledgebank that will collect and present new insights to pregnant and breastfeeding women and their HCP.
